# The American Association of Obstetricians and Gynecologists

**Published:** 1891-10

**Authors:** 


### THE AMERICAN ASSOCIATON OF OBSTETRICIANS AND GYNECOLOGISTS.

THIS hard-working medical organization has recently held its fourth annual meeting, and the proceedings will be published in the abstracts of the *American Journal of Obstetrics* for October.

The attendance was good, and the scientific work it accomplished was eminently satisfactory. The President, Dr. A. H. Wright, of Toronto, in his address gave a résumé of the purposes of the organization, and especially its negotiations with the Congress of American Physicians and Surgeons with reference to membership in that body. We wish we had space to print it in full, for it will be found full of interest to many.

The New York Academy of Medicine, where the meeting was held, on the 17th, 18th, and 19th days of September, is a splendid building, and the Association was treated most handsomely by the officials that controlled the edifice. A banquet was held on Friday evening, Sept. 18th, at which forty covers were laid, and the following toasts were given: The American Medical Association, responded to by Dr. Henry O. Marcy, of Boston ; The American Gynecological Society, response by Dr. E. H. Grandin, of New York ; The American Association of Obstetricians and Gynecologists, response by Dr. L. S. McMurtry, of Louisville ; The New York Academy of Medicine, response by Dr. Daniel Lewis, of New York ; *The American Journal of Obstetrics*, response by Dr. Paul F. Mundé, of New York; The Dominion Medical Societies, response by Dr. Adam H. Wright, of Toronto. This dinner was given in the banquet room of the Academy, which is a most delightful place for such a feast. Taken all together, the fourth annual meeting of this Association has demonstrated the vitality and strength of the organization in a remarkable degree. It now numbers over eighty members, composed of active, progressive, and competent men, who are making themselves felt in the professional world.

Dr. A. Vander Veer was elected President for the ensuing year, and the next meeting will be held in St. Louis, in September, 1892.

